# Integrative analysis of multi‐omics data reveals the heterogeneity and signatures of immune therapy for small cell lung cancer

**DOI:** 10.1002/ctm2.620

**Published:** 2021-12-19

**Authors:** Yabin Chen, Zhaoyuan Fang, Ying Tang, Yujuan Jin, Chenchen Guo, Liang Hu, Yang Xu, Xidong Ma, Jie Gao, Mei Xie, Xuelei Zang, Sanhong Liu, Haiquan Chen, Roman K. Thomas, Xinying Xue, Hongbin Ji, Luonan Chen

**Affiliations:** ^1^ State Key Laboratory of Cell Biology Shanghai Institute of Biochemistry and Cell Biology Center for Excellence in Molecular Cell Science Chinese Academy of Sciences Shanghai China; ^2^ School of Life Science and Technology ShanghaiTech University Shanghai China; ^3^ University of Chinese Academy of Sciences Beijing China; ^4^ Zhejiang University‐University of Edinburgh Institute Zhejiang University School of Medicine Haining China; ^5^ Department of Respiratory and Critical Care Chinese PLA General Hospital Beijing China; ^6^ Department of Pathology Chinese PLA General Hospital Beijing China; ^7^ Department of Radiology Affiliated Zhongshan Hospital of Dalian University Dalian China; ^8^ Clinical Laboratory Chinese PLA General Hospital Beijing China; ^9^ Institute of Interdisciplinary Integrative Medicine Research Shanghai University of Traditional Chinese Medicine Shanghai China; ^10^ Department of Thoracic Surgery Fudan University Shanghai Cancer Center Shanghai China; ^11^ Department of Oncology Shanghai Medical College, Fudan University Shanghai China; ^12^ Department of Translational Genomics Center of Integrated Oncology Cologne‐Bonn Medical Faculty University of Cologne Cologne Germany; ^13^ Department of Respiratory and Critical Care Beijing Shijitan Hospital Capital Medical University, Peking University Ninth School of Clinical Medicine Beijing China; ^14^ School of Life Science Hangzhou Institute for Advanced Study University of Chinese Academy of Sciences Hangzhou China; ^15^ Key Laboratory of Systems Biology Hangzhou Institute for Advanced Study University of Chinese Academy of Sciences Hangzhou China; ^16^ Center for Excellence in Animal Evolution and Genetics Chinese Academy of Sciences Kunming China

Dear Editor,

Small cell lung cancer (SCLC) features with high heterogeneity and poor prognosis. The major treatment strategy for SCLC remains combination chemotherapy over past decades, with only minor improvements.^1^ And the application of immunotherapy has no impressive benefit for SCLC patients.^2^ In this study, we identify a novel subtype of SCLC (SCLC‐I) with immunosuppressive feature and high genomic instability. Importantly, we find that POU2F3 is effective in predicting SCLC‐I, and the POU2F3‐high patients exhibit better responses to immunotherapy.

We performed weighted correlation network analysis (WGCNA)^3^ to explore heterogeneity of SCLC through integrating transcriptomic data detailed in the Supplementary Material (SM), including 19 Chinese surgical samples and 112 samples from two public data.^4^, ^5^ We firstly transformed gene expression data into co‐expression module to capture genes with similar expression pattern and extracted 17 coherent modules. All SCLC samples were clustered into four subtypes (Figure 1A), which showed no obvious difference in survival (Figure 1B). Each subtype showed comparable percentages in these three datasets (Figure 1A, Figure S5A), indicative of equal contribution of these datasets in the integrative analysis.

**FIGURE 1:**
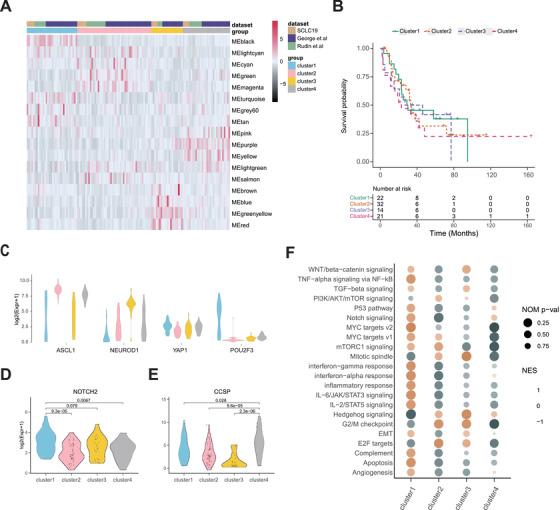
Subtyping 129 human small cell lung cancer (SCLC) using weighted gene co‐expression network. (A) Heatmap of eigenvectors of 17 network modules from weighted correlation network analysis (WGCNA). We combined three datasets after removing the batch effect by ComBat from ‘sva’ package in R. We divided the network into modules according to the correlations between the genes. The minimum size of the module was set as 10. We obtained 1016 genes for calculating adjacency matrix (power = 3). The co‐expression network was finally partitioned into 17 modules. All samples were divided into four clusters according to the module eigengenes by hierarchical clustering analysis (Euclidean distance, ward.D2 linkage). (B) Survival analysis of four SCLC clusters. (C) Violin plots indicating the range of expression of four markers (ASCL1, NEUROD1, YAP1 and POU2F3) among different clusters. (D) Violin plot of NOTCH2 level in each cluster. (E) Violin plot of CCSP level in each cluster. (F) Gene set enrichment analysis (GSEA) with normalized enrichment score (NES) and Nominal *p*‐values for hallmark gene sets associated with four clusters. The brighter color of NES indicates the up‐regulation of the pathway

To compare our findings with the previous study, in which SCLC was classified into SCLC‐A, SCLC‐N, SCLC‐P and SCLC‐Y,^6^ we evaluated the relative expression of these markers in our clusters. We observed that cluster 2 and cluster 3 corresponded to the SCLC‐A and SCLC‐N, respectively (Figure 1C). Consistent with the observation from Gay et al.,^7^ we found that YAP1 had low expression and did not exclusively define a subtype of SCLC (Figure 1C). We found that CCSP (SCGB1A1), a secreted protein mainly produced by club cells to maintain airway integrity,^8^ was specifically highly expressed in cluster 4 (Figure 1E, Figure S3E), indicating that certain SCLC might originate from club cells. Hereafter, we named cluster 4 as SCLC‐C. The cluster 1 showed low ASCL1 and NEUROD1 expression and high POU2F3 and NOTCH2 expression (Figure 1C,D). Gene set enrichment analysis (GSEA) demonstrated that the interferon‐gamma response, interferon‐alpha response, inflammatory response and IL‐6/JAK/STAT3 signaling were enriched in SCLC‐I subtype (Figure 1F), which clearly confirmed its immune‐related characteristic. We therefore named cluster 1 as 'immune subtype (SCLC‐I)'.

Further analysis showed that the enrichment of immune‐related pathways, such as IL‐10 signaling and other interleukin‐related pathways, in SCLC‐I versus other subtypes (Figure 2A, Figure S4B). Multiple immuno‐inhibitory factors such as PD1, IL‐10, IDO1, CD96 and BTLA, were highly expressed in SCLC‐I (Figure 2B,E, Figure S4). Immune cell infiltration is considered to be primary immune signature and strongly associated with the clinical outcome of cancer immunotherapies.^9^ Using ImmuCellAI,^10^ we found that the abundance of dendritic cells, macrophage, induced regulatory T cells (iTreg) and CD8^+^ T cells were significantly increased in SCLC‐I (Figure 2C). Moreover, we observed that most of the chemokines, such as CXCL10, CCL17, CCL18, were up‐regulated in SCLC‐I subtype (Figure 2D). Taken together, SCLC‐I subtype is featured with the activation of immune checkpoint molecules and infiltration of immune suppressive cells such as iTreg, which might help the immune escape.

**FIGURE 2:**
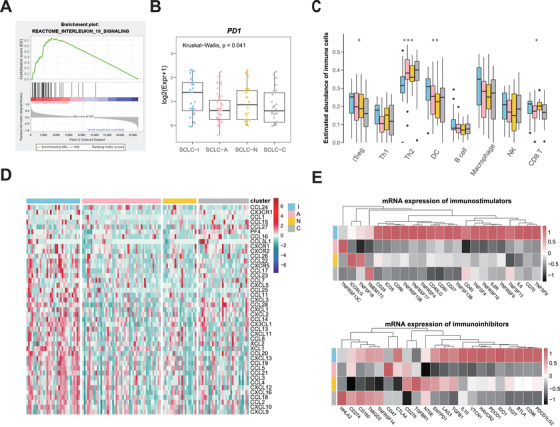
Immune signatures in the four small cell lung cancer (SCLC) subtypes. (A) Representative gene set enrichment analysis plot showing the enrichment of IL‐10 signaling pathway in SCLC‐I subtype. (B) Boxplots of PD1 expression in each cluster. (C) Relative abundance of indicated immune cell types in each cluster (calculated using the ImmuCellAI algorithm). The Kruskal‐Wallis test was conducted and annotated as follows: ***p < 0.001, **p < 0.01, *p < 0.05. (D) Heatmap of chemokines expression in each cluster. Chemokines are secreted proteins promoting immune cell infiltration and play an important role in tumour microenvironment. (E) The mean mRNA levels of immunostimulators (up) and immunoinhibitors (bottom) in each cluster. Annotated colors represent four subtypes (Skyblue = SCLC‐I, Pink = SCLC‐A, Gold = SCLC‐N, and Grey = SCLC‐C)

To explore the unique genetic alterations of each subtype, we performed analyses of 115 samples with available genomic sequencing data and RNA‐sequencing data. We found that each subtype harbored specific gene mutations, for example, LRP1, CD163, MME, ABCB1 mutations were frequently observed in SCLC‐I (Figure 3A). In comparison with other samples, SCLC‐I group showed significantly higher genetic alterations (Figure 3B,C), which indicated the increased genomic instability. SCLC‐I had more gene amplifications on Chr2, Chr6, Chr11, Chr12 and Chr19, whereas SCLC‐A had more gene amplifications on Chr17, SCLC‐N had more amplified genes on Chr9 and Chr21, and SCLC‐C had more amplified genes on Chr14 (Figure 3E, Table S2). Over 3000 genes were observed significantly amplified with high alteration frequency (>50%) in SCLC‐I (Figure 3D, Table S2). Further analysis showed that the cholesterol biosynthesis I pathway was significantly enriched in SCLC‐I subtype (Figure 3F). Consistent with the GSEA result using gene expression profile (Figure 1F), the WNT/beta‐catenin signaling was enriched in both SCLC‐I and SCLC‐N subtypes. Collectively, these data show that SCLC‐I subtype has higher level of genomic instability and each subtype harbors unique gene mutations and copy number variations.

**FIGURE 3:**
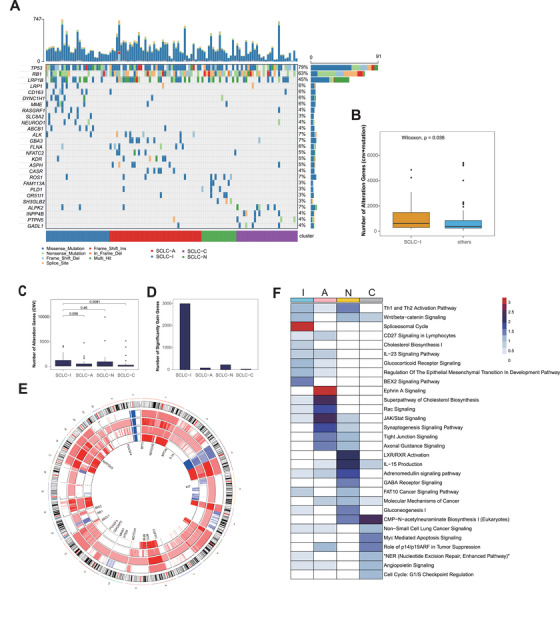
Copy number alterations and mutations in the four small cell lung cancer (SCLC) subtypes. (A) Top bar graph shows the number of nucleotide substitution mutations in each patient. Tumour samples are arranged from left to right according to different subtypes. Genes with high mutation rate or significantly mutated in each subtype (prop.test) are shown in waterfall plot. (B) Total number of gene alterations (mutations, gene amplifications and deletions) in SCLC‐I versus the rest of SCLC specimens. (C) Number of copy number alteration genes (sum of amplified and loss genes) of all clusters. (D) Number of significant gain genes (*p* < 0.05) with high alteration frequency (>50%) in four subtypes. (E) Correlation network analysis (CNA) heatmap across all chromosomes using median copy number in each subtype. We replace copy number as follows: −2 <= 0, −1 <= 1, 0 <= 2, 1 <= 3, 2 <= 4 and greater. From outside to inside, there are SCLC‐I, SCLC‐A, SCLC‐N and SCLC‐C. Some significant alteration genes in each subtype are highlighted in the inner ring. (F) Pathway enrichment analysis (IPA) using significant amplified genes with high genetic alteration frequency (freq > 50%, *p* < 0.001 for SCLC‐I, *p* < 0.05 for others) in each subtype. Heatmap is colored by '−log10(*p*‐value)'

To determine whether our findings have clinical relevance, we then identified the biomarker for SCLC‐I. According to feature selection from random forest model, we found that 10 genes (POU2F3, ANXA1, LRMP, GFI1B, SLC7A14, PHYHIPL, MAP2, SYP, KCNK3 and CPE) were most important in distinguishing SCLC‐I from other subtypes (Figure 4A, SM 6 and Figure S6A). Using these genes to build random forest model on 100 sets of different testing data, we got the average prediction accuracy of 92.74% and average score of area under curve (AUC) of 93.32% (Figure 4B, Table S3). Among 10 genes used for SCLC‐I prediction, POU2F3 stood out as the most significantly up‐regulated gene with high expression (Figures 4C and 1C and Table S5). Moreover, the SCLC‐P samples identified in the previous study^6^ were all included in SCLC‐I with up‐regulated immune‐related pathways (Figure S6C).

**FIGURE 4:**
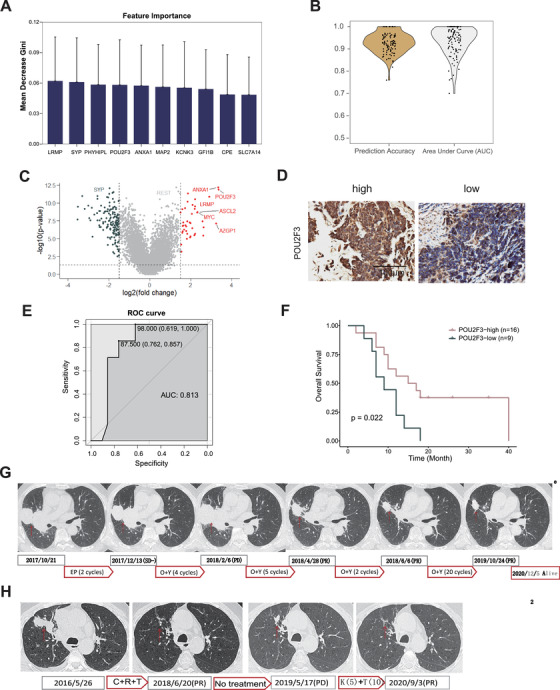
Patients with high POU2F3 levels respond well to second‐line immunotherapy. (A) Importance of the 10 features from 100‐times random sampling trainings. We built a random forest classifier based on the transcriptomic data to predict small cell lung cancer (SCLC)‐I subtype. We firstly performed 100‐times random samplings to divide training data and testing data. We chose 1000 genes with the highest coefficient of variation from training data to build each model. Then, we obtained the gene list ranked by Gini index. We counted the top 200 genes obtained each time among the 100 results from the 100 sets of training data. Finally, we got these 10 genes appeared more than 60 times. (B) Prediction accuracy and area under curve (AUC) scores using the 10 features over 100 sets of different testing data. (C) Volcano plot showing differentially expressed genes in SCLC‐I versus the other subtypes. (D) Representative POU2F3 immunohistochemical (IHC) staining in SCLC biopsy specimens. (E) Receiver operating characteristic (ROC) curve of POU2F3 IHC staining score corresponding to diagnosis of objective response rate (ORR). (F) Overall survival curves of patients with high or low POU2F3 levels. *p* value was calculated by Kaplan–Meier analysis with log‐rank test. (G) Representative photos of CT image of patient sjt27 at different time points during the whole treatment. The current clinical guideline for SCLC immunotherapy is 4–6 cycles, and the medication stopped if tumour progressed. We here prolonged the duration of immunotherapy to 8–10 cycles and found the final clinical outcome was effective, although some patients had pseudo progression after 4–6 cycles treatment. EP: carboplatin/etoposide. O+Y: Opdivo+Yervoy. (H) Representative photos of CT images of patient sjt22 at different time points during the whole treatment process. C+R+T: carboplatin+radiotherapy+temozolomide. K+T: Keytruda+temozolomide

We further collected a cohort containing 28 relapsed SCLC samples from patients receiving immunotherapy or chemo‐immunotherapy (Table S4, SM 7) and performed immunohistochemical staining of POU2F3 in these specimens (Figure 4D, Table S4). Our data showed that the patients with high POU2F3 expression exhibited a significantly improved objective response rate (ORR) to immunotherapy, with a high AUC of 0.813 (Figure 4E). Moreover, the POU2F3 protein level was positively correlated with patient prognosis (Figure 4F). Importantly, two patients with high POU2F3 level showed dramatic regression of lung tumours (Figure 4G,H). The positive response to immunotherapy indicated the potentially strong immune cell infiltration in POU2F3‐high SCLC. These results together supported that the SCLC‐I patients are more sensitive to immunotherapy, and POU2F3 might serve as a biomarker for SCLC immunotherapy.

In conclusion, our work systematically uncovers the transcriptomic and genomic heterogeneity in SCLC and characterizes a novel immune subtype with high sensitivity to immunotherapy. We identified POU2F3 as a potential biomarker with a good prediction power to assess SCLC immunotherapy response. Gay et al. identifies an inflamed subtype of SCLC which shows a significant overall survival (OS) benefit relative to all other subtypes with the combined chemotherapy and immunotherapy.^7^ In our study, the immune subtype seems correspond to a combination of SCLC‐P and SCLC‐I from Gay et al. cohorts. Although we used a different method and biomarkers to identify this special subtype of SCLC, both our study and Gay et al. study have proven the potential of SCLC re‐clustering in current clinical immunotherapy. Of course, the small size of the validation cohort is a limiting factor. Future clinical efforts and larger cohorts are required to validate the effectiveness of immunotherapy in this subtype.

## CONFLICT OF INTEREST

The authors declare that the research was conducted in the absence of any commercial or financial relationships that could be construed as a potential conflict of interest.

## Supporting information

Supporting information

TableS1

TableS2

TableS3

TableS4

TableS5
