# Supplementary material for: Integrative analysis of multi‐omics data reveals the heterogeneity and signatures of immune therapy for small cell lung cancer
Source: Clin Transl Med. 2021 Dec 19;11(12):e620. doi: 10.1002/ctm2.620 (PMC8684774; doi:10.1002/ctm2.620)
Supplement: Supplementary file 1 — Supporting information [file CTM2-11-e620-s003.docx]

**Supplementary material (SM)**

**Integrative analysis of multi-omics data reveals the heterogeneity and signatures of immune therapy for small cell lung cancer**

Yabin Chen^1,2,3,#^, Zhaoyuan Fang^4,#^, Ying Tang^1,3,#^, Yujuan Jin^1,3,#^, Chenchen Guo^1,3,#^, Liang Hu^1,3^, Yang Xu^5^, Xidong Ma^5^, Jie Gao^6^, Mei Xie^7^, Xuelei Zang^8^, Sanhong Liu^9^, Haiquan Chen^10,11^, [Roman K Thomas](https://pubmed.ncbi.nlm.nih.gov/?term=Thomas+RK&cauthor_id=26168399)^12^, Xinying Xue^13,5,*^, Hongbin Ji^1,2,3,14*^, Luonan Chen^1,2,15,16,*^

^1^ State Key Laboratory of Cell Biology, Shanghai Institute of Biochemistry and Cell Biology, Center for Excellence in Molecular Cell Science, Chinese Academy of Sciences, Shanghai 200031, China

^2^ School of Life Science and Technology, ShanghaiTech University, Shanghai 201210, China

^3^ University of Chinese Academy of Sciences, Beijing, China

^4^ Zhejiang University-University of Edinburgh Institute, Zhejiang University School of Medicine, Haining 314400, China

^5^ Department of Respiratory and Critical Care, Chinese PLA General Hospital, Beijing 100853, China

^6^ Department of Pathology, Chinese PLA General Hospital, Beijing 100853, China

^7^ Department of Radiology, Affiliated Zhongshan Hospital of Dalian University, Dalian, 116001, China

^8^ Clinical Laboratory, Chinese PLA General Hospital, Beijing 100853, China

^9^ Institute of Interdisciplinary Integrative Medicine Research, Shanghai University of Traditional Chinese Medicine, Shanghai, 201203, China

^10^ Department of Thoracic Surgery, Fudan University Shanghai Cancer Center, Shanghai, 200032, China

^11^ Department of Oncology, Shanghai Medical College, Fudan University, Shanghai, 200032, China

^12^ Department of Translational Genomics, Center of Integrated Oncology Cologne-Bonn, Medical Faculty, University of Cologne, 50931 Cologne, Germany

^13^ Department of Respiratory and Critical Care, Beijing Shijitan Hospital, Capital Medical University; Peking University Ninth School of Clinical Medicine, Beijing 100038, China

^14^ School of Life Science, Hangzhou Institute for Advanced Study, University of Chinese Academy of Sciences, Hangzhou 310024, China

^15^ Key Laboratory of Systems Biology, Hangzhou Institute for Advanced Study, University of Chinese Academy of Sciences, Chinese Academy of Sciences, Hangzhou 310024, China

^16^ Center for Excellence in Animal Evolution and Genetics, Chinese Academy of Sciences, Kunming 650223 China

* Corresponding authors: Luonan Chen (lnchen@sibs.ac.cn), Hongbin Ji (hbji@sibcb.ac.cn) or Xinying Xue (xuexinying2988@bjsjth.cn)

# These authors contributed equally to this work.

CONTENTS

[1 Human SCLC specimens 1](#_Toc31592)

[2 Processing of whole-exome sequencing (WES) data 1](#_Toc18842)

[3 Processing of RNA-seq data 2](#_Toc2066)

[4 Weighted gene co-expression network analysis (WGCNA) 2](#_Toc5748)

[5 Gene set enrichment analysis (GSEA) 3](#_Toc32541)

[6 Classification Model 3](#_Toc30242)

[7 Validation of POU2F3 in SCLC specimens 4](#_Toc30917)

[8 Supplementary Figures 6](#_Toc2483)

[9 Reference 16](#_Toc23702)

### 1 Human SCLC specimens

The 19 SCLC surgical specimens with paired pathological normal lungs were collected from July 2007 to June 2011 with the approval by the institutional review board of Fudan University Shanghai Cancer Hospital, Shanghai, China. All participants gave written informed consent. Fresh surgical specimens were snap-frozen and stored in liquid nitrogen upon resection until use. The pathology of each tumor sample was determined by pathologists. All these specimens were with a minimum of 70% of tumor cellularity. DNA was extracted from fresh-frozen tissues using the Gentra Puregene DNA extraction kit (Qiagen) following the protocol of the manufacturer. Total RNA was extracted from tissues with Trizol Reagent (Invitrogen) according to the manufacturer's instruction. The whole-exome sequencing was done in all of these specimens and their matched normal tissues, and RNA-seq was carried out in 19 tumor specimens and 5 normal specimens. All sequencing reactions were performed on an IlluminaHiSeq 2500 platform (Berry Genomics Corporation, China). The sequence mode is 125PE. Each sample produces no less than 8 GB clean data of whole exome sequencing, with all Q30 > 83%. In addition, we downloaded other two published SCLC datasets^1, 2^ with RNA-seq (George dataset = 81 tumors, Rudin dataset = 31 tumors), gene mutation and CNA data (George dataset = 110 tumors, Rudin dataset = 30 tumors). There are 158 samples with both gene mutation data and CNA information. Gene expression data and WES data of 19 Chinese SCLC patients are available via ENA Database under the accession number: PRJEB42904.

## 2 Processing of whole-exome sequencing (WES) data

Raw sequencing data was aligned to the human genome (GRCh38/hg38) using Burrows-Wheeler Aligner (BWA mem, version: 0.7.15)^3^. The data was then processed following the GATK (version: 3.5)^4^ best practices workflow to process the data. We marked duplicates with Picard (version: 2.0.1), performed local realignment around the indels (GATK RealignerTargetCreator) and recalibrated the base quality score (GATK BaseRecalibrator). Mutation calling was carried out by Mutect2 (DbSNP version 144 and COSMIC version v76). ANNOVAR^5^ was applied to the annotation of somatic mutations. Mutational landscape of 171 samples from three datasets was completed by R package ‘maftools’^6^.

Genome-wide copy number analysis was performed using CNVKIT (version: 0.9.6)^7^. For consistent with published two dataset of copy number, we calculated mean copy number of each gene from the output result of CNVKIT. CNA heatmap was completed by R package ‘RCircos’^8^. In Rudin’s cnv dataset, missing values were filled with the mean cnv values of each cluster. To present heatmap, we replace copy number as follows: -2 <= 0, -1 <= 1, 0 <= 2, 1 <= 3, 2 <= 4 and greater. Gene median copy number of each cluster was used to present gene alteration on all chromosomes.

## 3 Processing of RNA-seq data

Raw sequencing data was aligned to the human genome (GRCh 38/hg38) using STAR (version: 2.5.2b)^9^ 2-pass mode. Read counts were calculated by HTSeq (version: 0.6.0)^10^. FPKM was calculated by Cuffdiff (version: 2.2.1)^11^ for co-expression network construction.

## 4 Weighted gene co-expression network analysis (WGCNA)

The weighted gene co-expression network analysis was carried out by R package ‘WGCNA’^12^. The expression data for network construction from 129 samples after deletion of two outliners, including 19 patients, and another 80 patients and 30 patients collected from public datasets. We combined three datasets after removing the batch effect by ComBat from ‘sva’ package^13^ in R (Figure S3A). Genes with a low signal value (Mean Expression <0.3) or a low coefficient of variation (CV<1) were filtered out. We divided the network into modules according to the correlations between the genes. The minimum size of the module was set as 10. We obtained 1016 genes for calculating adjacency matrix (power = 3). The co-expression network was finally partitioned into 17 modules. We clustered the samples according to the module eigengenes by hierarchical clustering analysis (Euclidean distance, ward.D2 linkage). The 129 samples were finally clustered into 4 subgroups. The correlations between modules and subgroups were measured by Pearson correlation coefficient.

For analysis of the 110 samples from the public datasets, the minimum size of the module was set as 10. We obtain 1020 gene for calculating adjacency matrix (power = 5). The co-expression network is finally partitioned into 19 modules. We obtain four new clusters: cluster 1 (21), cluster 2 (36), cluster 3 (26), and cluster 4 (27). Major subtypes could be reproduced without 19 samples, especially SCLC-I (Supplementary Fig. 4)

## 5 Gene set enrichment analysis (GSEA)

GSEA was performed using the GSEA software (version:4.1.0) available at <http://software.broadinstitute.org/gsea/downloads.jsp> with the option pre-ranked and default parameters^14^. Reactome gene sets, KEGG gene sets and hallmark gene sets were used in the molecular signature analysis. We used NES and NOM p-value to present up-regulated pathways in each subtype.

## 6 Classification Model

We used random forest model to select features to identify the subgroup of SCLC patients. We performed 100-times random samplings to divide training data and testing data, and chose few common features as final one. We compared outcomes under different conditions. Specifically, in binary classification to only identify immune subtype, we firstly chose 1000 genes with the highest coefficient of variation from training data to build random forest. Then, we obtained the gene list ranked by Gini index. We counted the top 200 genes obtained each time among the 100 results from the 100 sets of training data. Finally, we got 10 genes (ANXA1, CPE, GFI1B, KCNK3, LRMP, MAP2, PHYHIPL, POU2F3, SLC7A14, SYP) appeared more than 60 times. Using these genes to build random forest model on 100 sets of testing data, we got the average prediction accuracy and the average AUC.

For module-based features, we firstly calculated differential expressed genes from each training data according to p-value of Kruskal-Wallis test among 1000 genes with the highest coefficient of variation. After auto-modeling by random forest, we got top 500 genes ranked by Gini index and built Pearson Correlation Coefficient network of these genes. We used (1-correlation) as distance for hierarchical clustering (ward.D2 linkage) and divided the 500 genes into 20 modules. Mean gene expression values of those modules were used as input to build decision tree (mtry = 2, ntree = 500). Top 1 module ranked by Gini index from 100 times is conserved (1×100). Here, different modules with over 50% gene duplication are regarded as the same one. We chose seven modules as final feature, which are non-repetitive and appeared more than 3 times. This network-clustering method can find genes with similar expression and stable relationship. Using these modules-based features to build random forest model on 100 sets of testing data, we got the average prediction accuracy. We also used another independent dataset consisting of 79 SCLC patients^15^ to test the model. Machine learning model was completed by R package ‘randomForest’^16^.

## 7 Validation of POU2F3 in SCLC specimens

A total of 28 SCLC patients were recruited from January 2016 to January 2020 with the approval by the Clinical Trial Ethics Committee of Beijing Shijitan Hospital (sjtky11-1x-2020(74)). Patients gave written informed consents. All methods were carried out in accordance with relevant guidelines and regulations. Biopsy samples were taken before clinical treatment. Twenty-seven patients received at least two cycles of chemotherapy until drug resistance with disease progression. Then, we gave them immunotherapy (Ipilimumab, Nivolumab, Carrilizumab (SHR-1210), Atezolizumab, Durvalumab) or chemo-immunotherapy (EP+Nivolumab, EC+Pembrolizumab etc.) (Supplementary Table 4). One patient was given EGFR-TKI (Icotinib) as first-line treatment and then received immunotherapy when drug resistance occurred. The clinical response was assessed after at least two cycles (21 days per cycle, the dosage followed in drug manufacture). Assessments of the clinical response were performed by the Response Evaluation Criteria in Solid Tumors (RECIST), version 1.1 (RECIST 1.1). According to RECIST v1.1, CR is the disappearance of any intratumoral enhancement in all target lesions. PR is at least a 30% decrease in the sum of maximum diameters of the enhanced tumor area. PD is at least a 20% increase in the sum of maximum diameters of the enhanced tumor area. We here defined SD(+) is less than 20% increase in the sum of maximum diameters of the enhanced tumor area, and SD(-) is less than 30% decrease in the sum of maximum diameters of the enhanced tumor area. After immunotherapy, 3 patients showed PD, 14 patients showed PR, 4 patients showed SD(+), and 7 patients showed SD(-). We collected survival prognostic information of 25 patients. Three patients were loss of follow-up.

For IHC staining, slides were de-paraffinized in xylene, and rehydrated with decreasing ethanol in water. Antigen retrieval was performed by microwave (high 5 min, low 25 min) in sodium citrate (pH 6.0) buffer. Slides were quenched in hydrogen peroxide (3%) to block endogenous peroxidase activity and washed in PBS buffer. Then incubated with POU2F3 antibody (NBP2-88074, Novus Biologicals, dilution 1:100) at 4℃ overnight. The IHC staining was blindly scored as high or low according to staining density and subjected to analyses for clinical relevance described previously^17^, and IHC score value of 90 was set as the cutoff to separate samples into “POU2F3 high” (IHC score >=90) and “POU2F3 low” (IHC score < 90) groups.

## 8 Supplementary Figures


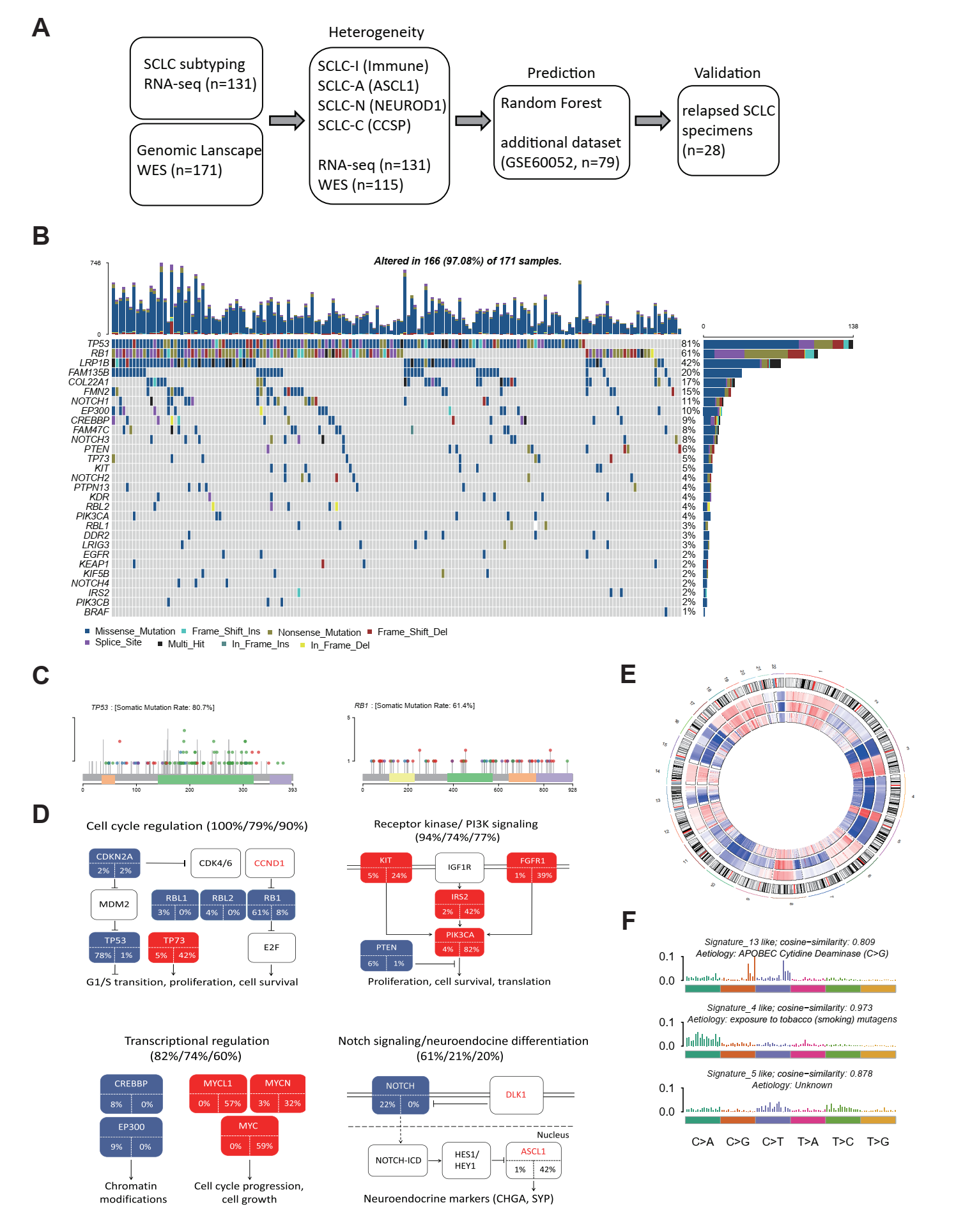


**FIGURE S1.** **The Genomic landscape of 171 human small cell lung cancer specimens.** (**A**) Schematic flowchart of analysis steps in this study. (**B**) Known cancer-related genes that were mutated in at least 2% of the cases in 171 samples. (**C**) Schematic representation of 2 candidate genes (TP53, RB1) with high mutations in respective protein domains. Somatic mutations and genomic translocations are mapped to the respective protein regions. Hotspot mutation types are highlighted in different colors. (**D**) Four major deregulated pathways in human SCLC. A total of 158 samples with both CNV and mutation data were used to calculate alteration proportions. Mutation ratios of each pathway in three datasets were shown in brackets (George dataset/our dataset/Rudin dataset). Red and blue boxes denote genes with activating and inactivating alterations, respectively. Gene mutation ratios are on the bottom left corner of each colored box. Gene amplification ratios are on the right bottom right corner of red boxes, and gene loss ratios are on blue boxes. Genes found expressed at high levels are shown in red font. (**E**) CNV heatmap using mean copy number in each dataset. From outside to inside, there are George dataset, Rudin dataset and our dataset (Chinese SCLC). (**F**) Three mutational signatures of specific pattern of nucleotide substitutions in SCLC.


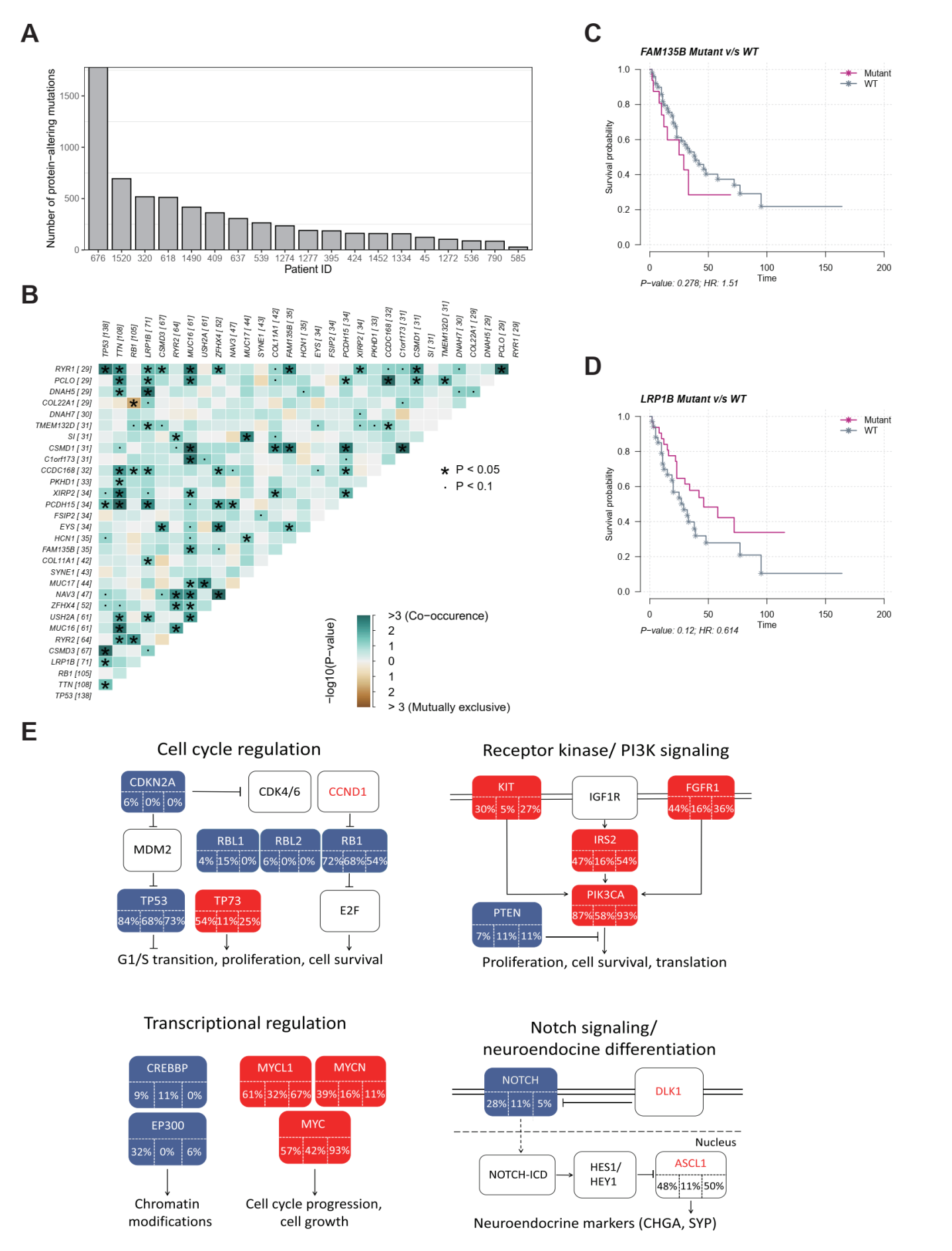


**FIGURE S2. Genomic characterization and analysis of human SCLC.** (**A**) Number of protein-altering mutations in 19 Chinese SCLC patients. (**B**) Mutually exclusive or co-occurring set of mutate genes in 171 human SCLC from three datasets (datasets from George et al., Rudin et al. and our 19 Chinese SCLC). Significant pairs of genes were marked as star using pair-wise Fisher’s Exact test. (**C**) Survival analysis based on LRP1B mutation status. (**D**) Survival analysis based on FAM135B mutation status. (**E**) Four signaling pathways recurrently deregulated in human SCLC. We calculated total frequency with mutation or copy number alteration of each gene in three datasets. From left to right in three boxes of each gene, there are George dataset, Rudin dataset and our dataset (Chinese SCLC).


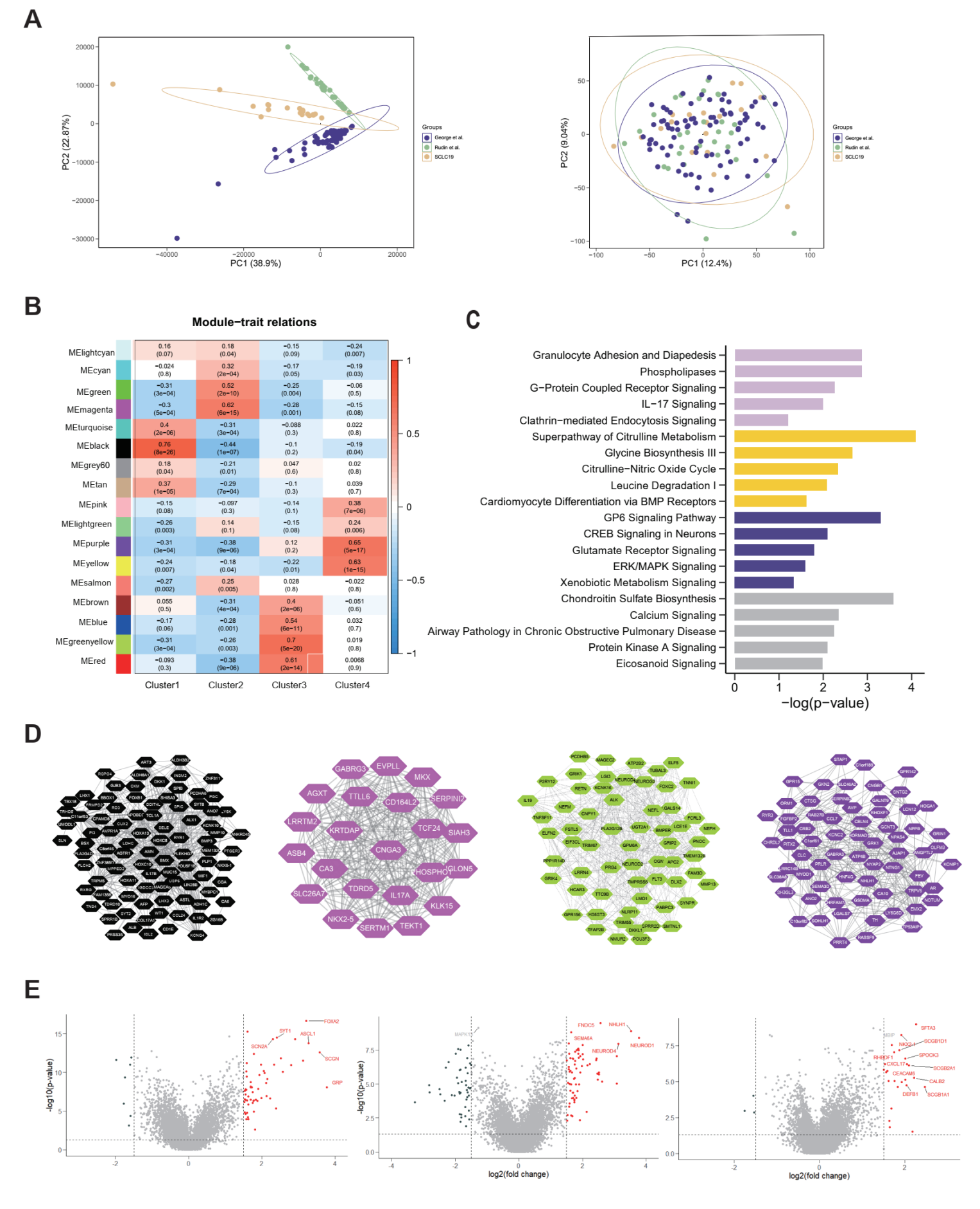


**FIGURE S3. Module-correlated analyses of human SCLC subtypes.** (**A**) PCA plot of three dataset (left). PCA plot of combined data after removing batch effect (right). (**B**) Heatmap of WGCNA module-trait relationship. The correlation between subgroups and modules was measured by Person correlation coefficient (PCC). (**C**) IPA canonical pathway analysis of genes in the most two correlated modules with each subtype. Annotated colors represent four subtypes (pink = cluster1, yellow = cluster2, blue = cluster3, gray = cluster4). (**D**) Gene co-expression network of MEblack, MEmagenta, MEgreenyellow and MEpurple, which corresponds to the most correlated traits of cluster1, cluster2, cluster3, and cluster4 respectively. (**E**) Volcano plot of differential expressed genes of one cluster versus others. From left to right, there are cluster2, cluster3, cluster4.


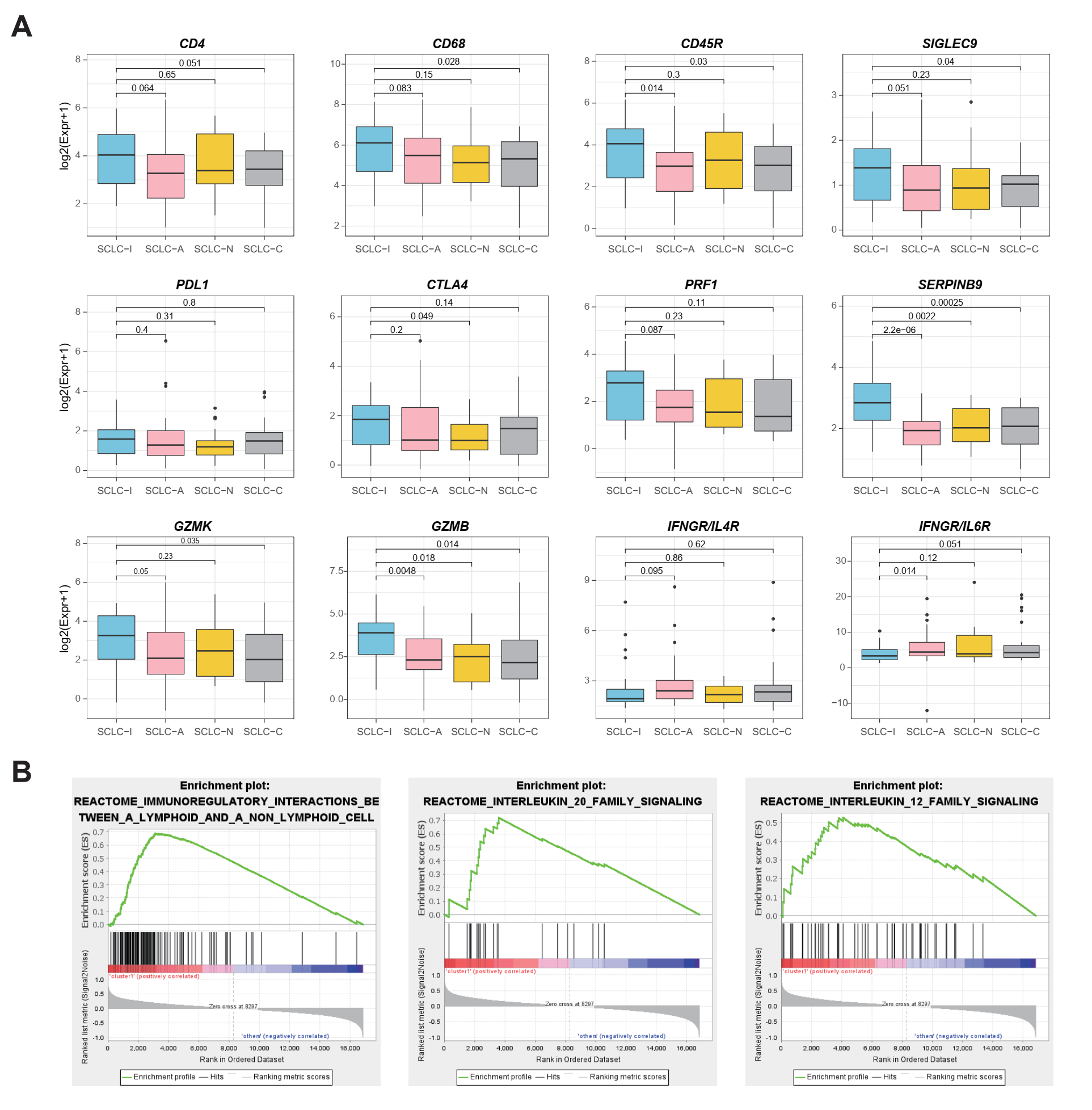


**FIGURE S4. Immune characteristics of human SCLC subtypes.** (**A**) Expression of immune related genes in four subtypes of SCLC. (**B**) Representative reactome genesets enriched in SCLC-I including Immunoregulation interactions between a Lymphoid and a non-Lymphoid cell, IL-20 Family signaling and IL-12 Family signaling.


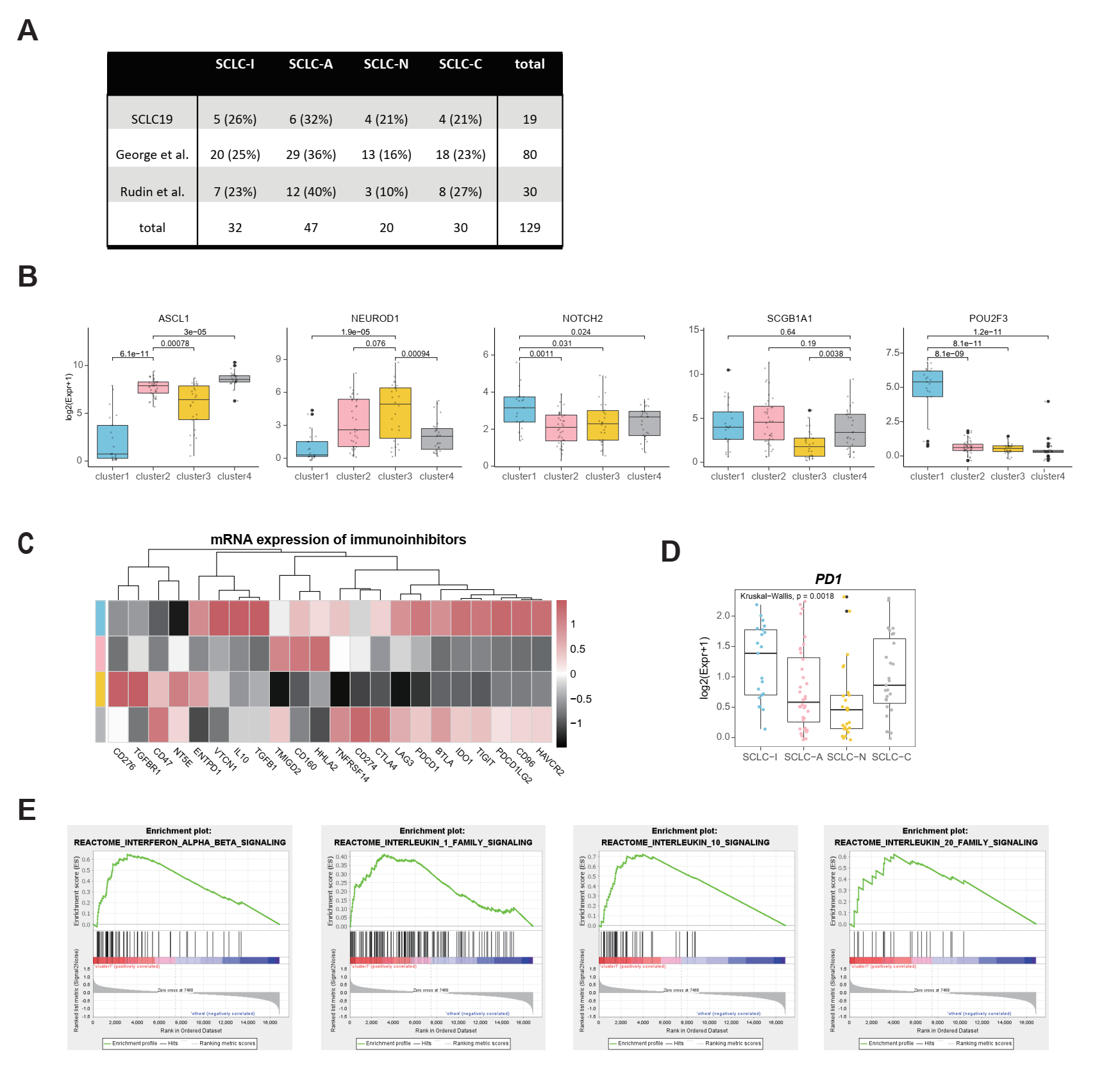


**FIGURE S5. Reproduced result of SCLC subtypes without 19 samples.** (**A**) Distribution of all the 129 SCLC from three datasets in four clusters. (**B**) Gene expression of five markers (ASCL1, NEUROD1, NOTCH2, CCSP, POU2F3) in four clusters. (**C**) The mean level of immune checkpoint gene transcription in four clusters. Annotated colors represent four subtypes. (**D**) Boxplot of PD1 gene expression in four clusters. (**E**) Representative reactome genesets enriched in cluster 1 including Interferon alpha/beta signaling, IL-1 Family signaling, IL-10 signaling and IL-20 Family signaling.


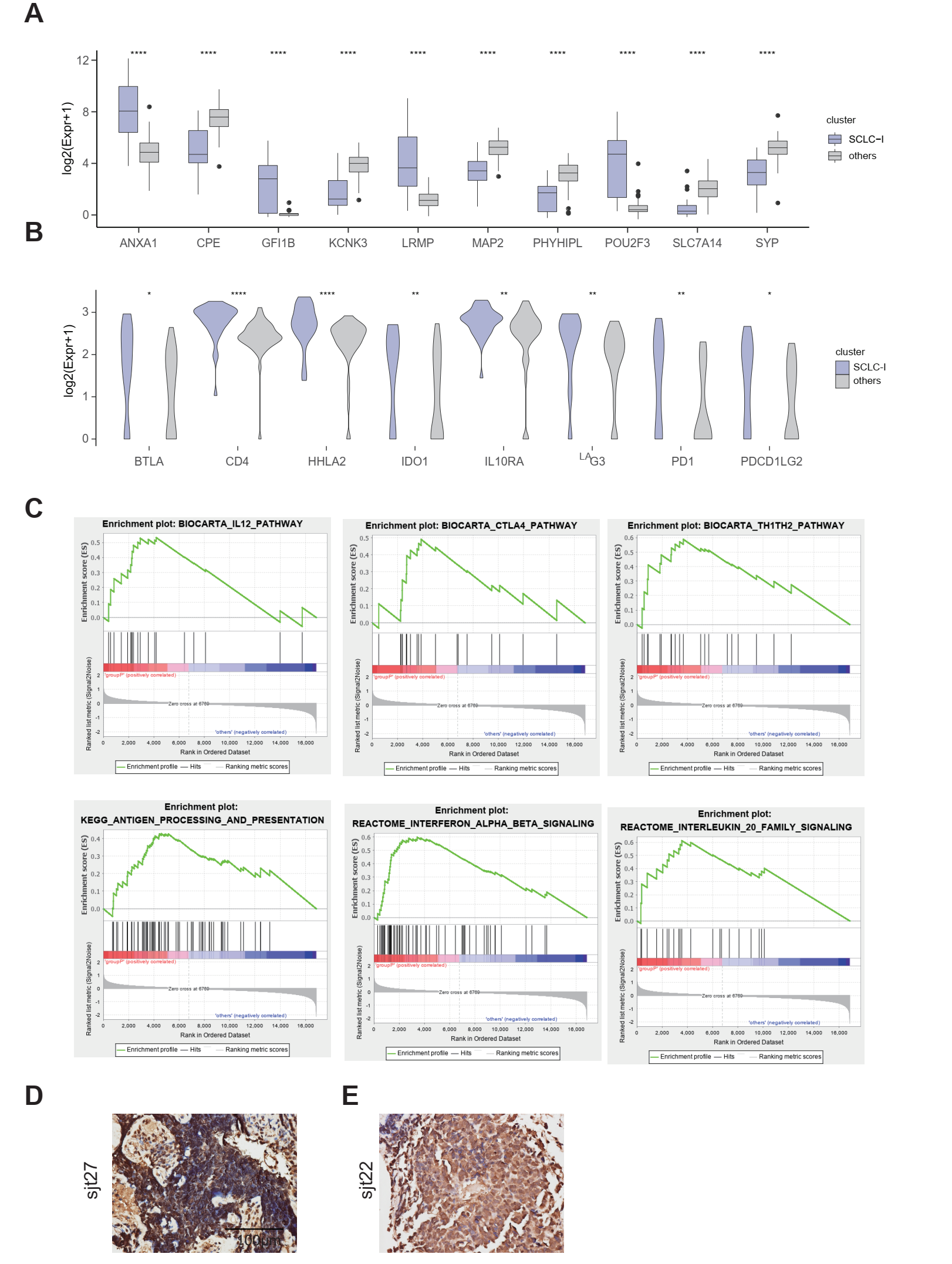


**FIGURE S6. Patients with high POU2F3 levels respond well to immunotherapy in second-line treatments.** (**A**) Expression of ten genes obtained from feature selection of random forest model in 129 SCLC samples. (**B**) Violin plots of the expression of eight immune checkpoint genes of the predicted SCLC-I subtypes and others from the independent SCLC samples (GEO Accession: GSE60052, SCLC-I = 33, others =46). (**C**) Gene set enrichment analysis plots of immune-related pathways in SCLC-P samples versus the rest samples in the public dataset from Nature Review (Rudin et al., 2019). (**D-E**) Photos of POU2F3 immunohistochemical staining in SCLC biopsy specimens from patient sjt27 (**D**) and sjt22 (**E**).

## 9 Reference

1. George J, Lim JS, Jang SJ, et al. Comprehensive genomic profiles of small cell lung cancer. *Nature* 2015;524:47-53.

2. Rudin CM, Durinck S, Stawiski EW, et al. Comprehensive genomic analysis identifies SOX2 as a frequently amplified gene in small-cell lung cancer. *Nat Genet* 2012;44:1111-1116.

3. Li H, Durbin R. Fast and accurate short read alignment with Burrows-Wheeler transform. *Bioinformatics* 2009;25:1754-1760.

4. McKenna A, Hanna M, Banks E, et al. The Genome Analysis Toolkit: a MapReduce framework for analyzing next-generation DNA sequencing data. *Genome Res* 2010;20:1297-1303.

5. Wang K, Li M, Hakonarson H. ANNOVAR: functional annotation of genetic variants from high-throughput sequencing data. *Nucleic Acids Res* 2010;38:e164.

6. Mayakonda A, Lin DC, Assenov Y, et al. Maftools: efficient and comprehensive analysis of somatic variants in cancer. *Genome Res* 2018;28:1747-1756.

7. Talevich E, Shain AH, Botton T, et al. CNVkit: Genome-Wide Copy Number Detection and Visualization from Targeted DNA Sequencing. *PLoS Comput Biol* 2016;12:e1004873.

8. Zhang H, Meltzer P, Davis S. RCircos: an R package for Circos 2D track plots. *BMC Bioinformatics* 2013;14:244.

9. Dobin A, Davis CA, Schlesinger F, et al. STAR: ultrafast universal RNA-seq aligner. *Bioinformatics* 2013;29:15-21.

10. Anders S, Pyl PT, Huber W. HTSeq--a Python framework to work with high-throughput sequencing data. *Bioinformatics* 2015;31:166-169.

11. Trapnell C, Roberts A, Goff L, et al. Differential gene and transcript expression analysis of RNA-seq experiments with TopHat and Cufflinks. *Nat Protoc* 2012;7:562-578.

12. Langfelder P, Horvath S. WGCNA: an R package for weighted correlation network analysis. *BMC Bioinformatics* 2008;9:559.

13. Leek JT, Johnson WE, Parker HS, et al. The sva package for removing batch effects and other unwanted variation in high-throughput experiments. *Bioinformatics* 2012;28:882-883.

14. Subramanian A, Tamayo P, Mootha VK, et al. Gene set enrichment analysis: a knowledge-based approach for interpreting genome-wide expression profiles. *Proc Natl Acad Sci U S A* 2005;102:15545-15550.

15. Jiang L, Huang J, Higgs BW, et al. Genomic Landscape Survey Identifies SRSF1 as a Key Oncodriver in Small Cell Lung Cancer. *PLoS Genet* 2016;12:e1005895.

16. Breiman L. Random forests. *Mach Learn* 2001;45:5-32.

17. Li F, Han X, Li F, et al. LKB1 Inactivation Elicits a Redox Imbalance to Modulate Non-small Cell Lung Cancer Plasticity and Therapeutic Response. *Cancer Cell* 2015;27:698-711.
